# Expanding Coordinated Specialty Care to Early‐Stage Bipolar Disorder: Development and Implementation of the STRIDE Model

**DOI:** 10.1111/eip.70155

**Published:** 2026-03-10

**Authors:** Kathleen Miley, Carissa Coudray, David Bond, Piper Meyer‐Kalos

**Affiliations:** ^1^ HeatlhPartners Institute Minneapolis Minnesota USA; ^2^ University of Minnesota Medical School Department of Psychiatry and Behavioral Sciences Minneapolis Minnesota USA; ^3^ Johns Hopkins School of Medicine Department of Psychiatry and Behavioral Sciences Baltimore Maryland USA

**Keywords:** bipolar disorder, early intervention, feasibility, first episode psychosis, psychoeducation, psychosocial therapies, recovery

## Abstract

**Background:**

Coordinated specialty care (CSC) is the gold‐standard early intervention model for schizophrenia‐spectrum disorders. Similar models for bipolar disorder (BD) are lacking. This paper describes the development and implementation of STRIDE, an adaptation of the NAVIGATE CSC model for early‐stage BD.

**Methods:**

Adaptations were developed based on literature review, international expert consultation and engagement with patient, family and clinician advisory groups. STRIDE was designed to be integrated with schizophrenia‐spectrum CSC services to build on existing expertise and infrastructure. Demographic and clinical data were collected at enrollment to characterise an initial STRIDE cohort of 19 participants between October 2023–June 2025.

**Results:**

A detailed model of CSC for BD was developed and implemented. Many components of NAVIGATE, including a recovery focus, shared decision making and the coordinated services of individual psychotherapy, medication management, family psychoeducation and supported education and employment were maintained. Key adaptations for BD included modification of individual psychotherapy modules to address prevention and treatment of mood episodes, addition of modules on circadian and social rhythms, affect regulation and managing common psychiatric comorbidities, development of an early‐stage BD prescriber's manual and tailored family psychoeducation. Of 19 patients in the STRIDE cohort (mean age 25 years, 52.6% female), 89.5% were diagnosed with bipolar 1 disorder and 94.7% had a history of psychosis. Anxiety, depression, functional impairment and sleep disturbances were commonly reported at baseline.

**Conclusions:**

STRIDE is a comprehensive adaptation of CSC for early‐stage BD. Further work is needed to assess STRIDE's feasibility, acceptability and effectiveness.

## 1 Introduction

Bipolar disorder (BD) is a common and disabling mood disorder which affects at least 37 million people worldwide (Ferrari et al. 2024). BD is the 4th leading cause of disability in young people (GBD 2019 Mental Disorders Collaborators 2022), driven by recurrent episodes of depression and mania/hypomania, which have devastating impacts on functioning and place patients at high risk for suicide (Plans et al. 2019; Simon et al. 2007). Clinical staging models conceptualise BD as a progressive condition beginning with an asymptomatic phase (stage 0), progressing to a first episode of threshold mood symptoms (stage 2) and for some individuals, multiple relapses and treatment resistance (stage 4) (Berk et al. 2007; Kapczinski et al. 2014). In line with the staging model, early diagnosis and treatment of BD are needed to prevent it from becoming more severe and disabling (Berk et al. 2010; Kozicky et al. 2016; Ratheesh et al. 2023; Swann et al. 1999). Some evidence suggests that pharmacological and behavioural treatments have greater efficacy when started early in the illness (Ratheesh et al. 2023), pointing to a potential critical period of intervention, consistent with the critical period hypothesis of psychosis (Birchwood et al. 1998). However, significant treatment delays exist, averaging up to 6–10 years and are associated with more time in mood episodes, psychiatric hospitalizations, self‐harm and functional decline (Buoli et al. 2021; Scott et al. 2022).

Young individuals with early‐stage BD have many treatment needs that must be addressed to achieve symptomatic and functional recovery. The onset of BD in the late adolescent to young adult years interrupts important developmental milestones such as accomplishing work, school and relationship goals (Coello et al. 2024; Sletved et al. 2022). Following an initial diagnosis of BD, relapse rates into mood episodes in those receiving medication treatment are 40% at 1 year and 60% at 4 years (Gignac et al. 2015b). Even after recovery from mood episodes, subthreshold mood symptoms and comorbid psychiatric conditions like anxiety disorders and ADHD are common and further impair functioning (Marangell et al. 2009; Perlis et al. 2006; Sentissi et al. 2008; Spoorthy et al. 2019; Tohen et al. 2003). Many other factors, such as developing psychological and behavioural symptom management strategies, avoiding substance use, regulating sleep, managing comorbid medical conditions and developing a support network are needed to help individuals with BD stay well and meet their life goals (Burdick et al. 2022; Gimenez‐Palomo et al. 2024).

Comprehensive BD early intervention programs to ensure early, accurate diagnoses and meet all of these needs are critically important to promote sustained recovery but are scarce. There is no defined standard of care for early‐stage BD and treatment guidelines are lacking (Chia et al. 2019). While some novel multidisciplinary early intervention programs for BD are emerging (Kessing et al. 2013; Ratheesh et al. 2025), most individuals are likely to receive treatment from fragmented clinical services in community settings from clinicians who are not specialists in BD. In contrast, a large evidence base has established coordinated specialty care (CSC) as the gold standard for individuals experiencing early‐stage schizophrenia spectrum disorders (SSDs) (Fusar‐Poli et al. 2017). CSC programs typically include up to 2 years of multidisciplinary services and have been shown to improve treatment engagement, psychiatric symptoms, hospitalisation rates, work and school participation and quality of life compared to usual care (Correll et al. 2018; Kane et al. 2016). NAVIGATE is a CSC model that was studied in the Recovery After an Initial Episode of Schizophrenia (RAISE) trial. NAVIGATE includes recovery‐focused psychotherapy, medication management, family education and school and education supports (Kane et al. 2016; Mueser, Penn, et al. 2015). RAISE demonstrated the effectiveness and feasibility of CSC in the United States health care landscape (Dixon et al. 2015; Kane et al. 2016), leading to the extensive implementation of schizophrenia‐spectrum CSC programs nationally (George et al. 2022).

There is an untapped opportunity to leverage the evidence base and infrastructure of schizophrenia‐spectrum CSC to develop and rapidly scale early intervention for BD. BD and SSDs are first diagnosed in young people and lead to many of the same developmental challenges and treatment needs. Yet, critical adaptations to schizophrenia‐spectrum CSC models are needed to address the unique clinical and functional needs of early‐stage BD. We developed STRIDE, an adaptation of the NAVIGATE CSC model, extensively tailored to early‐stage BD. STRIDE was designed to be integrated into existing early‐stage schizophrenia‐spectrum CSC teams to extend the reach of these services to those with BD. The purpose of this paper is to describe the development and intervention components of STRIDE, its initial clinical implementation and baseline characteristics and clinical profiles of the first cohort of enrolled patients. The feasibility, acceptability and preliminary outcomes of STRIDE will be described in a future report.

## 2 Methods

### 2.1 Intervention Development

STRIDE was developed by a team of investigators with expertise in early‐stage BD (DJB, KMM) and the NAVIGATE model for SSDs (PSM‐K, KMM) between November 2021 and June 2023. The Minnesota Department of Human Services funded its development due to a recognised need for early intervention in BD and to expand the capacity of NAVIGATE teams to better serve rural communities with a lower census of individuals with SSDs. We first brainstormed key needs of people with early‐stage BD that were not met by the existing NAVIGATE intervention, informed by extensive literature review. We then selected a group of internationally recognised experts in BD (*N* = 16) to provide feedback on the new or modified intervention components. To ensure STRIDE addressed all domains important to people living with BD and their families, we conducted 10 meetings with a patient advisory board of five BD patients and two meetings with family members and community clinicians. International experts with specific domain expertise worked with us to finalize the intervention components of STRIDE. Patients, family and community clinicians reviewed and contributed to select content areas included in the individual therapy and family psychoeducation modules, including a general overview of the STRIDE model and treatment components and specific feedback on intervention content spanning health and wellness, peer support, dialectical behaviour therapy (DBT), wellness planning and substance use. Patient and family advisors also contributed personal stories to be used in therapy modules focused on processing the first episode(s) for mania. STRIDE was so named by the patient advisory board to represent ‘hitting your stride’ and thriving with BD.

### 2.2 Target Population and Setting

STRIDE is designed for individuals aged 15–40 years who have received no more than 1 year of treatment for BD. The age range was chosen to align with the NAVIGATE inclusion criteria (Kane et al. 2016) and broadly captures the typical age of onset for bipolar spectrum disorders (Baldessarini et al. 2010; Bolton et al. 2021). It was developed to treat the entire range of bipolar‐spectrum disorders, including bipolar 1, bipolar 2 and other specified BD, with or without a history of psychosis. The program is intended to be implemented within existing NAVIGATE CSC teams in community settings. Integrating STRIDE within NAVIGATE CSC programs allows teams to build on existing expertise in providing recovery‐centred, team‐based care to young individuals with SMIs and leverages the existing infrastructure that supports team‐based care. Furthermore, the underlying diagnosis at enrollment into CSC is often not definitive. In situations where the diagnosis is not clear, decisions can be made about whether a patient should start with STRIDE or standard NAVIGATE based on team, patient and family consensus. Integration of STRIDE within a CSC program facilitates coordination or transition of care between STRIDE and NAVIGATE to best align with evolving clinical presentations. Like NAVIGATE, individuals may initiate services with STRIDE via referral from community clinicians, inpatient facilities or by self‐referral.

### 2.3 Clinical Program: Baseline Characteristics and Symptom Profiles

To assess the preliminary feasibility of extending NAVIGATE services to early‐stage BD, STRIDE was implemented as a pilot program in October 2023 at the University of Minnesota outpatient psychiatry clinic in a NAVIGATE‐informed CSC service. Demographic and clinical data were collected at enrollment from clinical interviews and electronic health records. Additional self‐report measures were completed via participation in the University of Minnesota's Early Psychosis Intervention Network (EPINET) study, EPI‐MINN. Self‐report measures included (1) the 10‐item Drug Abuse Screening Test (DAST‐10) (Skinner 1982), (2) the 3‐item Alcohol Use Disorders Identification Test (AUDIT‐C) (Barbor et al. 1992), (3) the 8‐item Patient Reported Outcomes Measures Information System (PROMIS) sleep disturbance short form (Yu et al. 2012) and (4) the Minnesota Symptom Severity Scale, which assesses the presence of a range of psychiatric symptoms and functional needs rated on a 0 (absent, not at all) to 4 (severe, nearly every day) scale. The University of Minnesota Institutional Review Board approved the implementation of STRIDE under a quality improvement designation and approved all EPI‐MINN activities. Patients enrolled in EPI‐MINN provided authorization to use their clinically‐obtained data for research purposes using an IRB‐approved form. Descriptive statistics were used to characterise the initial cohort of STRIDE participants who enrolled between October 2023–June 2025.

## 3 Results

### 3.1 Intervention Description

As with NAVIGATE, three conceptual frameworks, which are relevant to both young individuals with BD and SSDs, guided the development of STRIDE: the recovery model, the stress‐vulnerability model and psychiatric rehabilitation (Mueser, Gottlieb, et al. 2015). STRIDE is tailored to the unique needs and developmental stage of adolescents and young adults with BD. Treatment is aligned with goals related to individuation in relationships, vocational or educational pursuits, fostering self‐management of symptoms based on understanding the illness and individual risk and resiliency factors and facilitating self‐determination to counter the psychological impacts of living with a chronic illness (Mueser, Penn, et al. 2015).

Individuals with SSDs and early‐stage BD share many clinical characteristics already addressed by NAVIGATE, including high risk for problematic substance use (Duffy et al. 2014; Goldstein et al. 2013), suicidality (Simon et al. 2007) and decline in social and occupational functioning (Gignac et al. 2015a; Tohen et al. 2003). These components of NAVIGATE required no or limited adaptations for STRIDE. For example, the STRIDE Prescriber's Manual, like the NAVIGATE Prescriber's Manual (Robinson et al. 2018), emphasises shared decision‐making, improving adherence, ensuring medication tolerability and mitigating long‐term health risks and closely integrating with other team members and supports. STRIDE individual therapy maintains NAVIGATE's focus on psychoeducation, increasing resiliency and meeting personal goals, increasing natural supports and evidence‐based therapeutic skills such as cognitive‐behavioural therapy (Mueser, Penn, et al. 2015). Supported Employment and Education (SEE) services is a key NAVIGATE intervention that is transdiagnostic and was not modified for STRIDE (Mueser, Penn, et al. 2015).

However, STRIDE incorporates several key differences that are critical for recovery in BD (Tables 1 and 2). BD and SSD have different primary symptoms, different rates of comorbidities and distinct drivers of functional impairment. BD is predominantly a mood disorder and threshold and subthreshold mood symptoms, particularly depression and comorbid psychiatric illnesses like anxiety disorders and ADHD are the primary drivers of functional impairment (Burdick et al. 2022). In contrast, positive symptoms, negative symptoms and cognitive impairments predominate and drive poor functioning in SSDs (Santesteban‐Echarri et al. 2017). To address these differences, medication management, psychoeducation and therapeutic skills in STRIDE extend to managing the full range of symptoms in BD (depression, (hypo)mania, mixed episodes, subthreshold mood symptoms), addressing common negative schemas specific to BD (Ball et al. 2003; Macneil 2009), the addition of DBT skills with evidence for improving emotion regulation and addressing suicidality in BD (Goldstein et al. 2024; Jones et al. 2023), an emphasis on stabilising circadian and social rhythms (Frank et al. 1994; Goldstein et al. 2014; Hlastala et al. 2010; Inder et al. 2015) and management of key psychiatric comorbidities including anxiety, posttraumatic stress symptoms and ADHD (Léda‐Rêgo et al. 2024).

**TABLE 1:** Key adaptations of the STRIDE prescribers manual.

Prescribers manual		
Identified need	Adaptation	Rationale
Pharmacotherapy guidance for the management of mood episodes in early‐stage BD	Provided guidance for medication management of bipolar depression, mania, hypomania, mixed episodes, and maintenance treatment, including dosing recommendations and side effect management specific to early‐stage patients	NAVIGATE prescribing guidance focuses on SSD psychosis only (Robinson et al. 2018). There are significant differences in pharmacotherapy between BD and SSDs (Keepers et al. 2020; Yatham et al. 2018). Antipsychotics are the mainstay of treating SSDs while mood stabilisers are the treatment of choice for BD, necessitating further guidance for clinicians. While antipsychotics are commonly used in BD, the treatment goal is most frequently treating mood episodes rather than psychosis. Some antipsychotics have mainly antimanic effects and some have mainly antidepressant effects. Typical dose ranges differ from the treatment of FEP, especially when treating depression.
Differences in treating psychosis symptoms in BD	Provided guidance for treatment of psychosis symptoms specific to BD, including selection and duration of treatment of antipsychotics.	Psychosis symptoms are common in early‐stage BD (Gignac et al. 2015a; Tohen et al. 2003), but their short‐ and long‐term treatment differs compared to SSD psychosis. Psychosis symptoms in BD occur only during mood episodes and are typically shorter in duration than in SSD psychosis. Treating and preventing psychosis in BD requires treating and preventing mood episodes. Tapering adjunctive antipsychotics after 6 months of stability in BD patients may promote better cognition and functioning, and reduce the risk of metabolic side effects, without increasing the risk of relapse (Ratheesh et al. 2023). Some evidence suggests that lithium may be more effective in preventing psychosis than an antipsychotic during maintenance treatment in early‐stage BD (Berk et al. 2017). Some antipsychotics may increase risk for depression in BD and should be avoided in patients with a predominantly depressive illness course (Yatham et al. 2018).
Pharmacotherapy recommendations for common psychiatric comorbidities in BD	Included sections on the treatment of anxiety disorders and ADHD for people with early‐stage BD.	Anxiety disorders and ADHD are two of the most common psychiatric conditions comorbid with BD and are associated with significant inter‐episode distress and functional impairment (Léda‐Rêgo et al. 2024; Pavlova et al. 2015; Sandstrom et al. 2021). Common medications for anxiety (e.g., SSRIs) and ADHD (e.g., stimulants) can precipitate mania and mood cycling in BD, requiring a careful balance between treating comorbidities and not worsening the course of BD.
Promoting utilisation of lithium	Provided a rationale for lithium as the gold‐standard maintenance treatment in BD, along with detailed guidance on dosing, monitoring, management of side effects, and mitigating health risks such as thyroid and renal impairment.	Lithium is widely considered the gold‐standard maintenance treatment for BD, and is one of the few medications with evidence for efficacy specifically in early‐stage BD (Ratheesh et al. 2023). It has robust evidence for treating and preventing depression and mania and may reduce suicide, premature mortality from cardiovascular disease and other medical conditions, and the structural and functional brain changes characteristic of BD (Baldessarini and Tondo 2000; Chen et al. 2023; Hafeman et al. 2012; Smith and Cipriani 2017; Yatham et al. 2018). Nonetheless, prescribers must be conversant with monitoring for long‐term side effects and be aware of toxicity in overdose. Rates of lithium prescription continue to decline (Malhi et al. 2023), suggesting a need for increased prescribing guidance.
Considerations for polypharmacy	Striving for monotherapy is a core treatment principle in first episode SSDs. While monotherapy is also ideally the goal for early‐stage BD, polypharmacy is often needed to adequately treat two types of mood episodes, comorbid psychiatric illnesses and sleep disturbances (Amerio et al. 2021). STRIDE focuses on treating patients with the fewest medications and lowest doses consistent with wellness, and preferential use of evidence‐based medications to reduce polypharmacy.	Pharmacotherapy for BD is complex and involves effectively treating and preventing depression without worsening mania, treating and preventing mania without worsening depression, and adequately addressing psychiatric comorbidities and sleep disturbances (Yatham et al. 2018). Only a small number of medications are effective in treating both depression and mania, and even fewer have robust maintenance data (Yatham et al. 2018). If psychiatric comorbidities continue to cause distress or impairment once euthymia has been achieved, they may require independent treatment. Guidance is provided on options for treating them without destabilising mood.
Promoting healthy sleep patterns	Medication and behavioural recommendations for treating sleep disturbances in mood episodes and during euthymia are provided.	Early‐stage BD patients have high rates of circadian rhythm sleep disturbances and sleep disorders. This is especially problematic because poor sleep can precipitate mania and depression and can worsen cognitive impairments and functioning. Promoting healthy sleep patterns is thus an important goal of BD pharmacotherapy (Melo et al. 2017).
Health promotion	Provided guidance on monitoring for and treating cardiovascular disease risk factors especially elevated BMI and tobacco use.	People with BD have high rates of cardiovascular disease and risk factors, leading to 10–12‐year shorter lifespans compared to those without BD (Chan et al. 2022). CVD risk factors appear early, making early‐stage BD an ideal time to initiate health promotion to prevent poor long‐term health (Miley et al. 2023). Weight management and tobacco cessation are two of the most important interventions for health promotion in early‐stage BD (Miley et al. 2023). They require a multi‐pronged treatment approach involving medication and lifestyle considerations.
Addressing reproductive concerns	Provided guidance on reproductive and women's health concerns relevant to early‐stage BD, including contraceptive and preconception planning considerations, risks of teratogenicity, and risk and treatment of mood episodes during the perinatal period.	Individuals in STRIDE will be aged 15–40, in their peak reproductive years, and many will require counselling and potential treatment adjustments for reproductive concerns. Some core BD medications carry significant teratogenic risks. The perinatal period is a high‐risk time for mood episodes, and knowledge of reproductive and teratogenic risks of common BD pharmacotherapy is needed.

Abbreviations: BD, bipolar disorder; SSD, schizophrenia spectrum disorders.

**TABLE 2:** Key adaptations of the STRIDE increasing resiliency in life manual.

Increasing resiliency in life		
Identified need	Adaptation	Rationale
Mood episode‐specific psychoeducation	Updated module to provide education on the full range of symptoms associated with BD including depression, mania, hypomania, mixed episodes and rapid cycling, Information is also provided on coming to terms with the consequences of mania and depression.	NAVIGATE Individual Resiliency Training (IRT) manual did not include education about the full range of BD symptoms.
Managing stress and building resiliency	Modified the stress‐vulnerability model for BD to include information about important BD‐specific resilience factors including maintaining a healthy sleep–wake cycle and the importance of routines in maintaining mood stability. Substance use is covered similarly to NAVIGATE as it is a concern across early‐stage BD and first episode SSDs.	Updated the discussion of stress vulnerability model to include key factors associated with stress and established treatment targets in BD treatment. Evidence‐based treatment for BD has validated approaches that include sleep and schedule tracking with strong links to circadian rhythms (Goldstein et al. 2014; Hlastala et al. 2010; Inder et al. 2015).
Wellness plan tailored to mood episode prevention	Extended the wellness plan to address the common triggers of depression and mania/hypomania and strategies to quickly reduce emerging mood symptoms. Expanded the focus on somatic health and wellness including healthy diet, physical activity, avoiding substance use.	The NAVIGATE Wellness Plan did not provide accommodations to track different triggers and early warning signs for (hypo)mania and depression. The STRIDE Wellness Plan includes common triggers and options to individualise treatment strategies according to the nature of the mood episode (Rodrigues Cordeiro et al. 2023).
Cognitive‐behavioural strategies for addressing BD‐specific schemas and sleep problems	Added topics to address common unhealthy core beliefs in BD and to use cognitive restructuring for worries about sleep.	The IRL module Dealing with Negative Feelings provides a structure to teach and practice cognitive restructuring. Empirically supported BD treatment includes cognitive behavioural approaches for insomnia and dysfunctional cognitive schemas (Ball et al. 2003; Harvey et al. 2015; Macneil 2009).
Coping with common symptoms	Expanded content to address coping strategies for common symptoms of BD including mania/hypomania, depression, anxiety, psychosis, sleep and trauma symptoms. An assessment battery with recommended self‐report screening tools for anxiety disorders, substance use disorders, borderline personality disorder, eating disorder, ADHD and trauma is also included in STRIDE.	People with BD have high rates of comorbid diagnoses and can struggle with sleep problems and posttraumatic stress symptoms (Léda‐Rêgo et al. 2024). Behavioural coping strategies were added to the IRT Coping with Symptoms module to address BD symptoms and common problems (Deckersbach et al. 2016; Macneil 2009; Murray et al. 2011; Ozdel et al. 2021).
Stabilising circadian, interpersonal and social rhythms	Included content on managing sleep and social rhythms adapted from Interpersonal and Social Rhythms Therapy (Frank et al. 1994)	People with BD often experience disruptions in their biological and social rhythms that can be linked to mood instability (Frank et al. 2019; Melo et al. 2017). Psychoeducation and sleep and routine trackers were incorporated into IRL to reduce the risk of relapse and support long‐term mood stability (Goldstein et al. 2014; Inder et al. 2015).
Managing trauma symptoms	Included modules focused on management of trauma related symptoms, including cognitive restructuring for post‐traumatic stress disorder and the BREATHE approach (Mueser, Gottlieb, et al. 2015; Nishith et al. 2015).	There are high rates of trauma exposure and posttraumatic stress disorder (PTSD) in early‐stage BD patients (Otto et al. 2004). The BREATHE and cognitive restructuring approach to PTSD are evidence‐based approaches to the treatment of posttraumatic stress symptoms that included a sample of people with serious mental illness including BD (Mueser, Gottlieb, et al. 2015; Nishith et al. 2015). BREATHE materials and cognitive restructuring strategies for PTSD cognitions were included in IRL.
Dialectical behavioural therapy (DBT) skills for BD	Developed DBT‐informed modules for addressing suicidal ideation, emotion regulation and distress tolerance.	BD is associated with one of the highest rates of suicide attempts (Simon et al. 2007). DBT strategies are associated with significant reductions in suicidality and in rates of self‐harm (DeCou et al. 2019; Kothgassner et al. 2021; Linehan et al. 2006). The IRL manual includes a module of DBT strategies designed to reduce feelings of suicidality and self‐harm, reduce emotional instability, and reduce substance use, risky and addictive behaviours.

Abbreviation: BD, bipolar disorder.

### 3.2 STRIDE Treatment Team and Intervention Components

In line with NAVIGATE (Kane et al. 2016), STRIDE includes four core interventions delivered by a coordinated team of clinicians with training in early‐stage BD: personalised medication management guided by the STRIDE Prescriber's Manual, resilience‐focused individual therapy (Increasing Resiliency in Life; IRL), family psychoeducation and SEE services. Two additional recommended intervention components have been added: peer support and health promotion. A program director coordinates referrals and services and all clinicians meet weekly for individual treatment planning. By extending CSC to early‐stage BD, teams can better utilise their clinician resources, especially in rural areas with lower rates of SSDs and BD (Powell et al. 2021). For this pilot, existing CSC clinicians were utilised without adding additional staff time. While there is expected variation in staffing needs based on regional population characteristics and catchment area size, we describe estimated staffing needs for teams in the Supporting Information. Initial length of treatment for STRIDE is conceptualised as up to 3 years to allow treatment flexibility based on individual remission and recurrence patterns.

#### 3.2.1 Personalised Medication Management

STRIDE patients have frequent visits with a prescriber with training in the pharmacotherapy of early‐stage BD. The STRIDE Prescriber's Manual provides evidence‐based and expert‐informed guidance on pharmacotherapy for bipolar depression, mania, mixed states, maintenance treatment, common psychiatric comorbidities, side effect management, principles of health promotion and reproductive considerations. Due to the diagnostic instability of early‐stage BD patients, we also included a brief section on the pharmacological treatment of major depressive disorder with psychotic features. Initial medication doses and titration schedules are tailored to early‐stage patients. Principles of shared decision‐making (Farkas 2007; Towle et al. 1999) are followed and there is an emphasis on evidence‐based medications for BD, especially lithium (Ketter et al. 2006; Ratheesh et al. 2023; Swann et al. 1999; Yatham et al. 2018).

#### 3.2.2 Increasing Resiliency in Life (IRL)

IRL is a manualized individual therapy that supports patients in recovery goals, provides education about BD and its treatment, promotes resiliency, improves self‐management and helps patients develop tools to prevent and manage symptoms. Following the NAVIGATE model, IRL is a recovery‐oriented psychotherapy rooted in cognitive behavioural therapy, illness management and recovery and motivational enhancement (Mueser, Penn, et al. 2015). It comprises 16 skills‐focused modules spanning BD symptom management, stress and resiliency, healthy lifestyles, relapse prevention and planning, processing mood episodes, DBT skills for BD, addressing symptoms and problems associated with trauma, decreasing substance use, stabilising circadian and social rhythms and improving interpersonal skills. STRIDE IRL clinicians receive training in these evidence‐based treatments and tailor the duration or depth of each module to individuals' specific needs. Some content areas are integrated throughout several IRL manuals. For example, content on stabilising circadian and social rhythms is included in the modules on managing stress and building resiliency, healthy lifestyles, cognitive approaches to sleep and coping with sleep problems (see Table 2 and the Supporting Information for further IRL module descriptions). The typical duration is 6–12 months. IRL is delivered by masters‐level clinicians.

#### 3.2.3 Family Support & Education

STRIDE family supports aim to establish collaborative relationships between patients, their families and the treatment team, teach family members about BD and equip families with skills to assist their loved ones with their goals. Family education includes a 10–12 session curriculum covering BD symptoms, treatment and course; relapse prevention planning with family and patient; developing family resiliency; problem solving and communication strategies; and stress reduction skills. Family education is typically provided by the program director, a masters‐level clinician (Mueser, Penn, et al. 2015).

#### 3.2.4 Supported Education and Employment (SEE)

The goal of SEE is to assist patients in achieving their educational and employment goals. A SEE specialist, typically a bachelor's‐level clinician, engages the patient in goal setting and provides practical assistance in job and educational program searching, obtaining work‐ and school‐related accommodations and identifying strategies to improve work and school performance (Mueser, Penn, et al. 2015; Rosenheck et al. 2017).

#### 3.2.5 Additional Recommended Interventions

In addition to the standard NAVIGATE interventions, we recommend that STRIDE CSC teams include a peer support specialist who can meet individually with STRIDE patients. Peer support specialists can improve engagement, benefit recovery outcomes, reduce crisis service and inpatient admissions and increase self‐efficacy in people with SMIs (Fuhr et al. 2014; King and Simmons 2018; White et al. 2020). The peer specialist provides STRIDE patients with supports grounded in their own recovery journey and provides a valuable perspective to the treatment team. To address cardiometabolic health disparities faced by young individuals with BD (Chan et al. 2022; Miley et al. 2023), STRIDE includes a health promotion intervention which extends the health promotion content in the Prescriber's Guide and IRL therapy. It includes (1) a health promotion group curriculum drawn from evidence‐based interventions for BD with a focus on behaviour change and motivation enhancement for increasing physical activity and improving nutrition (Green et al. 2015; Sylvia 2015; Yarborough et al. 2016) and (2) recommendations for individual health coaching delivered by a registered nurse or health coach and individually tailored to assist patients with goal setting, treatment planning, accountability of lifestyle modifications and liaising with clinicians.

### 3.3 STRIDE Team Training

NAVIGATE clinicians receive extensive training in engaging and treating individuals with SSDs. To extend this expertise to early‐stage BD, additional training in BD assessment, course and treatment is needed. Recommended training includes an overview for all team members on the bipolar prodrome, pathways to care in BD, challenges to engagement in treatment and diagnostic considerations (including differential assessment between BD and non‐affective psychotic disorders and assessment of psychiatric comorbidities using recommended assessment scales provided in the program manual; see Supporting Information for more detail). To foster engagement of diverse populations, STRIDE includes an updated Assessment and Initial Goal setting module which includes questions to elicit information about individuals' background, race, ethnicity and cultural experiences and how they may or may not interact with illness. This assessment is intended to support the provision of culturally competent care, foster treatment alliance and reduce disengagement (Powell et al. 2021). Individual therapists, family clinicians and program directors attend a subsequent training providing an overview of BD education and treatment approaches in the IRL and family education manuals. Medication training for BD is divided into two sessions. The first training is attended by all team members and provides an overview of medications used to treat BD, common side effects, assessment and treatment of common psychiatric comorbidities and strategies to improve health monitoring and health promotion. Prescribers attend an additional training that provides a more detailed overview of the recommended medication guidelines. Following the training, two consultation call groups are provided, one for all team members and one for prescribers and team leaders, to discuss and problem‐solve any challenges.

### 3.4 Baseline Characteristics and Self‐Reported Measures

Nineteen patients enrolled in STRIDE between October 2023–June 2025 and provided authorization and consent for data collection. STRIDE patients had a mean age of 25 years and 52.6% identified as female (Table 3). At enrollment, 89.5% of patients were diagnosed with bipolar 1 disorder and 10.5% were diagnosed with bipolar 2 disorder or other BD. The most common mood state at enrollment was depressed (57.9%) (mean PHQ‐9 score 7.6 (SD 3.9)), with the remaining patients euthymic. Most patients (94.7%) had a history of psychosis. The mean number of medications prescribed was 2.5 (1.2), with 42.1% of patients prescribed 3 or more medications. 68.4% of patients were working or in school. The majority (73.7%) reported symptoms of at least one psychiatric comorbidity or substance use problem. Problematic drug use was self‐reported by 41.1% of patients, while 11.8% reported problematic alcohol use. Common self‐reported symptoms included anxiety (64.7%), depression (64.7%), difficulty functioning at work or school (58.8%) and sleep disturbance (41.2%). No patients reported current psychosis symptoms at enrollment.

**TABLE 3:** Demographics and clinical characteristics at enrollment (*N* = 19).

	Mean (SD) or *N* (%)
Age (years)	25.0 (5.1)
Female	10 (52.6%)
Race	
White	17 (89.5%)
Other^a^	2 (10.5%)
Level of education	
High School or less	4 (21.0%)
Some college or college degree	10 (52.6%)
Graduate degree	5 (23.7%)
Currently enrolled in school or employed	13 (68.4%)
Bipolar disorder diagnosis	
Bipolar 1 disorder	17 (89.5%)
Bipolar 2 disorder or other bipolar disorder	2 (10.5%)
History of psychosis	18 (94.7%)
Mood state at enrollment	
Depressed or mixed	11 (57.9%)
Manic	0 (0.0%)
Euthymic	8 (42.1%)
Medications	
Mood stabiliser	14 (73.17%)
Antipsychotic	15 (78.9%)
Antidepressant	4 (21.0%)
Other	7 (36.8%)
Total medications prescribed	2.5 (1.2)
3 or more medications prescribed	8 (42.1%)
1 or more clinically significant comorbid mental health or substance use concerns	14 (73.7%)
Problematic drug use^b^ (DAST‐10) (*N* = 17)	7 (41.2%)
Problematic alcohol use^c^ (AUDIT‐C) (*N* = 17)	2 (11.8%)
Mild–moderate or greater sleep disturbance (PROMIS Sleep Disturbance Short Form) (*N* = 17)	5 (29.4%)
Self‐reported symptoms^d^ (*N* = 17)	
Anxiety	11 (64.7%)
Depression	11 (64.7%)
Mania	2 (11.8%)
Psychosis	0 (0.0%)
School or work functioning problems	10 (58.8%)
Sleep problems	7 (41.2%)
PHQ‐9 depression total score (*N* = 17)	7.6 (3.9)
PHQ9 score 5–9 (mild depression)	10 (58.8%)
PHQ9 score ≥ 10 (moderate or greater depression)	4 (23.5%)
GAD7 anxiety total score (*N* = 13)	5.2 (4.5)
GAD7 score 5–9 (mild anxiety)	3 (23.1%)
GAD7 score ≥ 10 (moderate or greater anxiety)	3 (23.1%)

Abbreviations: GAD7, generalised anxiety disorder‐7; PHQ‐9, patient health questionnaire; PROMIS, patient reported outcome measures information system.

^a^ Due to small numbers of patients in other race categories or of Hispanic ethnicity, we only include two categories here.

^b^ Proportion scoring in the moderate level or greater range on Drug Abuse Screening Test (DAST‐10).

^c^ Proportion with positive screen on the Alcohol Use Disorders Identification Test (AUDIT‐C).

^d^ Proportion endorsing symptom category as occurring at least ‘several days’ in the past 2 weeks.

## 4 Discussion

In this report, we describe STRIDE, an adaptation of NAVIGATE CSC (Kane et al. 2016) for early‐stage BD and the baseline characteristics of the initial cohort of STRIDE patients. Our initial implementation and enrollment data show that patients with early‐stage BD will seek care in CSC programs. Our assessment of baseline characteristics of the initial STRIDE cohort suggests that the intervention components are aligned with identified clinical domains. Further evaluation of the feasibility, acceptability and effectiveness of STRIDE is needed.

STRIDE demonstrates that the NAVIGATE CSC model for SSD can be adapted for people with early‐stage BD. Several CSC intervention components and treatment tenets—including a focus on recovery and shared decision‐making, along with SEE services—are transdiagnostic and require little modification. Other intervention components required significant adaptations to adequately target the treatment needs of young individuals with BD. Key modifications addressed the full range of mood symptoms in BD (depression, (hypo)mania, mixed episodes), inter‐episode emotion regulation, negative schemas, suicidality and comorbidities including anxiety, PTSD, ADHD and circadian rhythm disturbances. Due to the extensive differences in prescribing for SSDs and BD (Robinson et al. 2018; Yatham et al. 2018), the STRIDE Prescriber's Manual was created anew, although a focus on shared decision‐making, promoting adherence and enhancing tolerability of medications was maintained.

Consistent with research in first‐episode mania cohorts (Gignac et al. 2015b), we identified many potential treatment needs in our early‐stage BD cohort. At enrollment, STRIDE patients tended to have threshold or subthreshold depression symptoms. A large portion had psychiatric or substance use comorbidities and polypharmacy was common. The most common self‐reported symptoms were depression, anxiety, sleep disturbances and impairment in psychosocial functioning. While STRIDE intervention components address each of these areas, longitudinal evaluation will be needed to understand how well STRIDE leads to improvements in symptoms and functioning.

Few evidence‐based, multidisciplinary early intervention programs for BD exist. Kessing et al. (2013) found that 2 years of care in a specialised mood disorder clinic in Denmark, including evidence‐based pharmacologic treatment and group psychoeducation, led to reduced hospitalizations, increased use of mood stabilisers and increased care satisfaction compared to treatment as usual. Analysis of outcomes at a 16‐year follow‐up suggested promising long‐term effects on suicidality and found that the intervention was associated with fewer (but not statistically significant) psychiatric admissions, lower costs and increased use of lithium and antipsychotics (Munkholm and Kessing 2024). Ratheesh et al. (2025) developed BLEND, a 6‐month early intervention program with components of guideline‐concordant pharmacotherapy, a blended in‐person individual therapy combined with digital therapeutic content and digital relapse monitoring. In a sample of 21 young people, BLEND was found to be feasible, acceptable and led to improvements in suicidality. STRIDE contains similar components to these models, but the specific team structure and integration with schizophrenia‐spectrum CSC distinguish STRIDE from existing early‐intervention models. Although digital interventions are an emerging area for early intervention, they are not yet standard of care in most CSC programs and incorporation of a digital component for STRIDE was not feasible during initial intervention development.

STRIDE was designed to be integrated with SSD CSC programs to leverage existing resources and clinical expertise in treating early‐stage SMIs. As such, it can be readily deployed and scaled as an extension of the more than 350 CSC programs in the United States, of which NAVIGATE is the most common treatment model (Kazandjian et al. 2022). However, integrating STRIDE with SSD CSC services may also present some challenges. First, it may be challenging to reach people experiencing BD without psychosis, as indicated by the high proportion of patients enrolled into STRIDE with a history of psychosis. The best way to reach people with BD without psychosis, including those with bipolar 2 disorder and other specified BDs, requires further study and may require active community outreach and engagement with existing mental health services. It is also unknown how the care pathways and service use of CSC interventions will differ among those with BD compared to SSDs (Phalen et al. 2025; Polillo et al. 2023). It is possible that the frequency of engagement and the length of care required to achieve recovery in BD differs from that in SSDs. For example, due to the episodic nature of mood episodes in BD, CSC for early‐stage BD may benefit from more flexible enrollment periods with the ability of patients to step up or down in service intensity or to return to services after discharge in the early illness phases, as needed. Additionally, functional impairments associated with early‐stage BD may differ compared to SSDs and may result in different SEE needs. Racial and socioeconomic disparities in access, engagement and outcomes associated with CSC have been identified (Bennett and Rosenheck 2021; Moe et al. 2024; van der Ven et al. 2022). In our initial STRIDE implementation, most enrolled patients were White and further efforts to understand how STRIDE can equitably reach those from diverse sociodemographic backgrounds are critical. There is currently no consensus definition of early‐stage BD and how to best operationalise this for clinical services like STRIDE remains an open question. Whether existing CSC teams have the capacity to extend their reach to those with BD is not completely understood. While equipping teams to treat early‐stage BD may enhance the sustainability of programs with lower census, such as those in rural areas, the demand for SSD CSC care is high and not fully met by current services in more populated areas (Kazandjian et al. 2022; Powell et al. 2021). The expansion of CSC to young individuals with BD will likely require support from healthcare funders to ensure access (Dixon 2017; George et al. 2022). We did not systematically collect STRIDE referral data to understand overall program demand and reach and such data would inform broader implementation feasibility. Finally, while CSC is the gold standard treatment model for first episode SSDs, it is unknown whether it is the optimal model for early‐stage BD and further research is needed to confirm whether the known benefits of CSC extend to those with early‐stage BD.

## 5 Conclusions

We developed and implemented STRIDE, an extension of the NAVIGATE CSC model for first episode SSDs tailored to early‐stage BD. By leveraging the extensive CSC infrastructure, STRIDE has the potential to be rapidly scaled in real‐world settings to fill a critical gap in early intervention for BD. Further research is needed to evaluate STRIDE's feasibility and effectiveness.

## Funding

This work was supported by the Minnesota Department of Human Services.

## Ethics Statement

The University of Minnesota Institutional Review Board approved implementation of STRIDE under a quality improvement designation and approved all EPI‐MINN study activities. Patients enrolled in EPI‐MINN provided authorization to use their clinically‐obtained data for research purposes using an IRB‐approved form. When a patient was under the age of 18, a parent or guardian co‐signed the authorization form; similarly, if the patient was an adult who had a legal guardian make medical decisions on their behalf, their guardian co‐signed. Analytic methods, study materials and data may be made available upon request to the corresponding author.

## Conflicts of Interest

Piper Meyer‐Kalos, Kathleen Miley and David Bond provide training and consultation about implementing STRIDE that can include compensation.

## Supporting information


**Data S1:** Supporting Information.

## Data Availability

The data that support the findings of this study are available from the corresponding author upon reasonable request.
