# Supplementary material for: Expanding Coordinated Specialty Care to Early‐Stage Bipolar Disorder: Development and Implementation of the STRIDE Model
Source: Early Interv Psychiatry. 2026 Mar 10;20(3):e70155. doi: 10.1111/eip.70155 (PMC12973362; doi:10.1111/eip.70155)
Supplement: Supplementary file 1 — Data S1: Supporting Information. [file EIP-20-0-s001.docx]

**Expanding Coordinated Specialty Care to Early-Stage Bipolar Disorder: Development and Implementation of the STRIDE Model**

**Supplemental Material**

**Recommended staffing of STRIDE**

For this pilot, existing CSC clinicians were utilized without adding additional full time equivalents (FTE). While there is expected variation in staffing needs based on regional population characteristics and catchment area size, we estimate that for a caseload of 35-40 STRIDE clients, teams will require the following full-time equivalents (FTE) of staff:

• Director: 0.5 FTE (often combined with family clinician role), a masters level clinician

• Family clinician: 0.5 FTE (often combined with director role), a masters level clinician

• Prescriber: 0.20 to 0.30 FTE, an M.D. or Nurse Practitioner or Physician’s Assistant

• Increasing Resiliency in Life (IRL) clinician: 1.0 FTE, a masters level clinician

• Supported Employment and Education (SEE) specialist: 1.0 FTE, an individual with a

bachelor’s level degree

Highly encouraged to be included as members of a STRIDE Team:

• Peer Specialist: 0.5 FTE, an individual with lived experience with mental health challenges

• Case Manager: 0.5 FTE, a person who meets the state and agency requirement to provide case management

• Health and Wellness coach (optional): 0.5 FTE, Mental health practitioner or registered nurse

For every additional caseload of 20-25 clients add:

• Increasing Resiliency in Life (IRL) clinician: 1.0 FTE, a masters level clinician

• Family clinician: 0.5 FTE (often combined with director role), a masters level clinician

• Supported Employment and Education (SEE) specialist: 1.0 FTE, an individual with a

bachelors level degree

**Summary of Increasing Resiliency in Life modules**

Please reference table below for comparison between NAVIGATE and STRIDE modules. Table includes synopsis of additions and revisions.

| Revisions by Module | | |
| --- | --- | --- |
| Navigate Modules IRT | STRIDE Modules IRL | Changes/revision/additions |
| Orientation   - Navigate program description - IRT Orientation - Guide to relaxed breathing | Orientation   - Navigate program description - IRL Orientation - Guide to relaxed breathing | Updated to include new STRIDE team members and additional IRL modules |
| Assessment/Initial Goal Setting   - Getting to know you - Identifying Strengths - Satisfaction with Areas of life - Identifying areas for improvement - Setting goals | Assessment/Initial Goal Setting   - Getting to know you - Identifying Strengths - Satisfaction with Areas of life - Identifying areas for improvement   Setting goals | Updated to include additional questions to elicit important information about individuals’ background, race, ethnicity, and cultural experiences and how they may or may not interact with illness |
| Education about Psychosis   - What is Psychosis? - Basic Facts about Alcohol and Drugs - Medications for Psychosis - Coping with Stress - Strategies to Build Resilience | Education about Bipolar Disorder   - Learning More about Your Experiences with Your Mood - What is Bipolar Disorder? How is Bipolar Disorder Diagnosed? | Updated the module to include the full range of symptoms associated with bipolar disorder including depression, mania, hypomania, mixed episodes, and rapid cycling. Also included information about the bipolar disorder diagnoses  included in STRIDE. |
|  | What Can You Do To Manage Stress and Build Resiliency?   - What is the stress- vulnerability model? - Medications for bipolar disorder - Basic facts about alcohol and drugs - Substance use and bipolar disorder - Coping with stress - What is the connection between mood and sleep? - Strategies to build resilience | Modified the stress- vulnerability model for bipolar disorder to include information about sleep and healthy routines. |
| Healthy Lifestyles   - Developing a healthy lifestyle - Getting active - Eating healthy - Making choices about smoking - Strategies for quitting smoking - Getting a good night’s sleep | Healthy Lifestyles   - Developing a healthy lifestyle - Getting active - Eating healthy - Making choices about smoking - Strategies for quitting smoking - Getting a good night’s sleep | Updated nutrition and exercise to include cognitive restructuring for addressing distressing thoughts related to making a lifestyle change.  Expanded the sleep topic to provide education on the biology of sleep, circadian rhythms, and monitoring of sleep schedules and habits. |
| Developing a Wellness Plan   - Developing a plan to stay well | Developing a Wellness Plan   - Developing a plan to stay well | Modified the Wellness Plan to address the common triggers of depression and mania/hypomania, individualized triggers for depression and  mania/hyomania. Included a focus on health and wellness |
| Processing the Psychotic Episode   - Telling your story - Challenging self- defeating thoughts and beliefs | Processing the Episode   - Telling your story - Challenging self- defeating thoughts and beliefs | Updated symptoms of bipolar disorder and common treatment experiences.  Adapted self-stigmatizing beliefs for bipolar disorder. Included a narrative from a young person with bipolar disorder and a writer and professor from Rutgers about her experience on the intersection of race and  bipolar disorder. |
| Developing Resiliency- Standard Sessions   - Exploring your resilience - Good things - Savoring - Mindfulness | Developing Resiliency- Standard Sessions   - Exploring your resilience - Good things - Savoring - Mindfulness | No significant changes |
| Building a Bridge to Your Goals   - Goal setting review - Moving ahead with a plan-Transitions in treatment | Building a Bridge to Your Goals   - Goal setting review - Moving ahead with a plan-Transitions in treatment | No significant changes |
| Dealing with Negative Feelings   - Taking charge of your Negative Feelings - Cognitive Restructuring for   Negative Feelings | Dealing with Negative Feelings   - Taking charge of your Negative Feelings - Cognitive Restructuring for   Negative Feelings   - Dealing with Distressing Core Beliefs - Cognitive Restructuring for Worries about Sleep | Added 2 topics to address common unhealthy core beliefs in bipolar disorder and to use the 5 steps of cognitive restructuring for worries about sleep. |
| Coping with Symptoms   - Identifying Symptoms that bother you - Coping with Anxiety - Coping with Depression - Coping with hallucinations - Coping with Sleep Problems - Coping with Low Stamina and Low Energy - Coping with Worrisome Thoughts | Coping with Symptoms   - Identifying Symptoms that bother you - Coping with Mania/Hypomania - Coping with Anxiety - Coping with Depression - Coping with hallucinations - Coping with Sleep Problems - Coping with Low Stamina and Low Energy - Coping with Worrisome Thoughts - Coping with   Posttraumatic Symptoms | Added 2 new topics to include coping strategies for mania/hypomania and postraumatic stress symptoms. Updated the coping strategies for sleep to expand with additional coping strategies based on common sleep problems associated with bipolar disorder. |
|  | Managing Your Symptoms of Trauma   - BREATHE –   Common Reactions to Trauma and Relaxed Breathing   - BREATHE –   Posttraumatic Stress Symptoms   - BREATHE –   Posttraumatic Stress Associated Problems   - Cognitive Restructuring for Posttraumatic Stress Symptoms | Added an evidence-based approach to the treatment of trauma for persons with serious mental illness including bipolar disorder. The topics provide education on the symptoms of posttraumatic stress disorder and associated problems.  Individuals also learn relaxed breathing to reduce overarousal. Lastly, individuals apply cognitive restructuring to posttraumatic stress cognitions. |
|  | DBT Skills for Bipolar Disorder   - Using Mindfulness to Improve Focus and Relaxation - Coping with Crisis - Emotion Regulation - Substance and Behavioral Addiction | New module created to address emotion  dysregulation, substance use comorbidity, and reduce crisis situations. |
| Substance Use   - Talking about substance use - Taking the first step: creating a healthy habit - Identifying areas of change and using strengths and resiliency - Trying it out: Having fun and socializing without using substances - Trying it out: Strategies to combat boredom - Trying it out: Strategies to deal with Negative feelings, symptoms, and sleep problems - Coping with cravings - Tools to help you make a change - Developing a plan to stay on track - Wrapping up and Looking to the Future | Substance Use   - Talking about substance use - Taking the first step: creating a healthy habit - Identifying areas of change and using strengths and resiliency - Trying it out: Having fun and socializing without using substances - Trying it out: Strategies to combat boredom - Trying it out: Strategies to deal with Negative feelings, symptoms, and sleep problems - Coping with cravings - Tools to help you make a change - Developing a plan to stay on track - Wrapping up and Looking to the Future | Added information about the interaction between substance use problems and mania/hypomania and strategies to help reduce substance use problems. |
| Having Fun and Developing Good Relationships   - Getting more fun in your life: Reviving previously enjoyed activities - Developing new fun activities - Getting the most out of your fun - Getting some practice talking with people - Re-connecting with old friends - Making new friends - Showing an interest in others - Improving communication with others - Managing Disclosure - Interpreting Social Cues | Having Fun and Developing Good Relationships   - Getting more fun in your life: Reviving previously enjoyed activities - Developing new fun activities - Getting the most out of your fun - Getting some practice talking with people - Re-connecting with old friends - Making new friends - Showing an interest in others - Improving communication with others - Managing Disclosure - Interpreting Social Cues | No changes |
| Developing Resiliency – Individualized Sessions   - Gratitude visits - Counting your blessings - Active/Constructive Responding - Life Summary - Practicing acts of kindness | Developing Resiliency – Individualized Sessions   - Gratitude visits - Counting your blessings - Active/Constructive Responding - Life Summary - Practicing acts of kindness | No changes |

**STRIDE recommended assessments**

The assessments included in the STRIDE Assessment Appendix are intended for use in routine care to help guide differential diagnosis and assessment of comorbid conditions common to bipolar disorder. Not all patients will require each measure.

| **Assessment** |
| --- |
| **Mood** |
| Mood and Feelings Questionnaire |
| General Behavior Inventory – 10 item mania form |
| General Behavior Inventory – 10 item Depression Form A |
| Hypomania Symptom Checklist (HCL-32) |
| **Anxiety** |
| Screen for Adult Anxiety Related Symptoms (SCAARED) |
| Screen for Anxiety Related Symptoms (Child Version): |
| **Attention Hyperactivity Deficit Disorder** |
| Adult ADHD self-report screening scale (ASRS-5) |
| **Personality disorders** |
| McLean Screening Instrument for Borderline Personality Disorder |
| **General distress** |
| Perceived Stress Scale (PSS) |
| **Trauma** |
| PTSD Checklist for DSM-5 (PCL-5) |
| Life events checklist |
| **Sleep** |
| PROMIS Sleep Disturbance Scale |
| PROMIS Sleep Related Impairment Scale |
| **Substance use** |
| CRAFFT |
| Drug Abuse Screening Test (DAST-10) |
| Brief Michigan Alcohol Screening Test (MAST-10) |
| **Suicide risk** |
| Columbia Suicide Symptoms Severity Rating Scale |
| **Obsessive Compulsive Disorder** |
| Yale Brown Obsessive Compulsive Scale (Y-BOCS) |
